# Food Reward and Distance Influence the Foraging Pattern of Stingless Bee, *Heterotrigona itama*

**DOI:** 10.3390/insects9040138

**Published:** 2018-10-11

**Authors:** Norasmah Basari, Sarah Najiah Ramli, Nur ‘Aina Syakirah Mohd Khairi

**Affiliations:** 1 School of Marine and Environmental Sciences, Universiti Malaysia Terengganu, Kuala Nerus 21030, Terengganu, Malaysia; sarahnajiah93@gmail.com (S.N.R.); elyakhazinatulyusra95@gmail.com (N.‘A.S.M.K.); 2 Center of Excellence Apis and Meliponine, Universiti Malaysia Terengganu, Kuala Nerus 21030, Terengganu, Malaysia

**Keywords:** foraging activity, food exploitation, sugar nectar concentration, tropical species, meliponiculture, bee farming

## Abstract

Beekeeping with stingless bee provides new opportunities to improve the incomes of many households in Malaysia through the sale of honey and other bee products. While *Heterotrigona itama* is one of the most commonly cultured species of stingless bees, its behavior is not very well understood. Hence, we conducted this study to investigate the behavior of *H. itama* in exploiting food sources by ascertaining the nectar sugar concentration preferred by the bee. We also aimed to determine the preferred distance of food source from the beehive. Our results suggest that *H. itama* prefers high sugar concentrations of 35% and above, and most of the bees preferred to forage close to their hive to collect food. We discuss how nectar concentration, food distance, and abiotic factors influence the number of bees exploiting food sources and the overall foraging pattern of *H. itama*.

## 1. Introduction

The stingless bee is a eusocial insect that belongs to the family Apidae and the tribe Meliponini [1,2,3]. The behavior of the stingless bee is dynamic in exploiting plant-based resources [4] such as nectar, pollen, resin, latex, leaves, scents, oil, and seed during their foraging flight [5]. Besides plant-based food resources, the stingless bee is also known to collect other sources, mainly for nest construction, such as animal feces, water, and clay [5]. Some species of stingless bees build their nests in cavities in pitted trunks or branches of living trees [6], mostly above 1 m from the ground, as well as in rock cavities [7]. There are at least 600 species of stingless bees in the world that are classified under about 60 genera, as compared with only 11 species of honeybees in a single genus, which is *Apis* [8]. There are many species of stingless bees that are still under study. Each species may have its own specific behavior and a requirement that needs to be understood not only to facilitate their culture for honey collection, but also to ensure the sustainability of the colonies, especially in the farming areas. 

Meliponiculture, or stingless beekeeping, has been gaining more attention in Malaysia recently. It provides new opportunities to increase household income, especially for rural people, through the sale of honey and other bee products such as bee bread and propolis. About 35 species of stingless bees (tribe Meliponini) have been identified in Peninsular Malaysia [9]. Among these, *Heterotrigona itama* and *Geniotrigona thoracica* are the most popular species in meliponiculture [10]. Previous studies have been carried out on the foraging behavior of various stingless bee species, for example *Hypotrigona gribodoi*, *Meliponula ferruginea* [11], *Melipona panamica* [12], and *Tetragonula biroi* [13]. However, the foraging behavior of *H. itama* remains largely unknown, and therefore, a comprehensive study in this respect was undertaken. With meliponiculture becoming an important economic resource for many households not only in Malaysia but also in other tropical countries [14,15], an intensification of the research on stingless bees to understand their foraging behavior is appropriate. This would help maximize honey production and also ensure that their colonies remain healthy.

Different species of stingless bee behave differently [16]; they have their own specific requirements and preferences in food resources. Some species may have a preference for nectar of different sugar concentrations. For example, *M. fasciata*, *M. compressipes triplaridis*, *M. fuliginosa,* and *M. marginata* collect flower nectar with sugar concentrations ranging from 21% to 60% [17]. *Hypotrigona gribodoi* has been recorded to prefer nectar with 14.2% to 67.4% sugar concentration, while *M. ferruginea* collects nectar with sugar concentrations ranging between 9.1–63.4% [11]. Another study on the sugar preference by *M. fasciata* revealed that this species might exploit flower nectar with 15% sugar concentration, but would switch to nectar of a higher sugar concentration (95%) when the latter was available [18]. In general, the concentration of sugar in nectar collected by the bees is between 35–65% [13,16].

Distance from the food source affects the foraging behavior of stingless bees. As the bees recruit foragers to an area that is rich with food sources [12], some species may show a significant preference toward food that is located at a certain distance. For example, Jarau et al. [1] found that *M. scutellaris* foraged 30 m from its nest, while *M. quadrifasciata* was found to be recruiting its nestmates from up to 40 m away. Other species such as *T. biroi* preferred to exploit food sources that were located closer (1 m) to their colonies [13].

Stingless bees may have one or two peak foraging hours, depending on species. For example, *Trigona iridipennis* has two peak hours of foraging activity, which are in the morning and in the afternoon [3], while the peak foraging hour for *Geotrigona subterranean* is during midday between 13:00–14:00 h [19]. Another stingless bee, *Plebeia remota,* carries out its peak foraging activities at different times according to the season. In its reproductive diapause, nectar foraging centers around noon, while it is around 10:00 h during its reproductive phase [5]. Foraging activity also strongly depends on abiotic factors. Previous studies revealed that foraging activity has a positive correlation with temperature and a negative correlation with relative humidity [20,21].

Since previous studies have shown that different species of stingless bees exhibit their unique behaviors in exploiting food sources, we conducted this study to examine how sugar concentration preference and distance from food source influenced the foraging pattern of *H. itama*. This study focused on helping beekeepers and farmers better arrange bee colonies and the food sources around the farming areas. Hence, the experimental set-up in this study was designed to be suitable for such areas. We are aware that the bees’ foraging behavior may be quite different under their natural environment where the food sources occurred naturally and scattered in the wild. 

## 2. Materials and Methods 

### 2.1. Study Site

**Figure 1:**
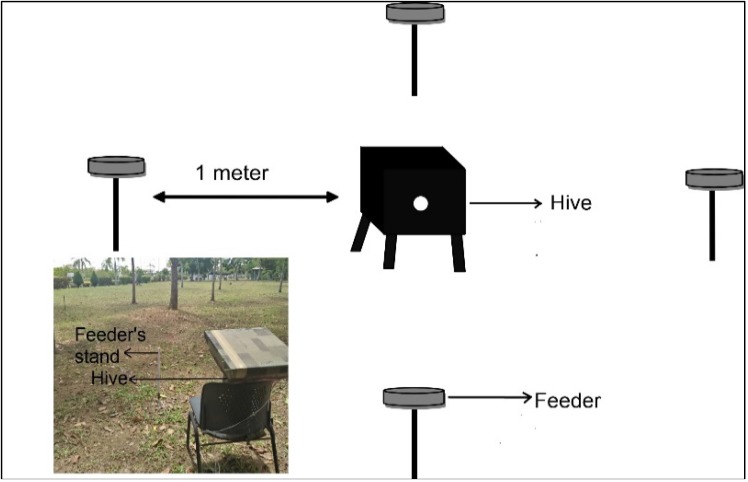
The experimental set-up for experiment 1. The set-up was a modified version from Ciar et al. [13]. Petri dishes contained water (0%), 15%, 35%, and 50% sugar (sucrose) concentration were placed around the colony according to the four cardinal directions: north, south, west, and east. The feeder stands were placed 1 m from the trial colony. The feeders were rotated clockwise every three hours. The white dot on the beehive represents the colony entrance (faced to the east during the actual experimental trials). Inset: the actual experimental area.

This study was carried out at Universiti Malaysia Terengganu (UMT), which is located in Kuala Nerus, Terengganu (5.406020 N, 103.086918 E). The experiments were conducted from January until May 2017 in an open area with the presence of few *Bucida* sp. plants (Figure 1). The plants were not in the flowering season during the experimental period. 

### 2.2. Experimental Design

Two experiments were conducted: (1) sugar concentration preference, and (2) foraging distance. The experimental set-ups for Experiment 1 and Experiment 2 were modified from the previous set-up by Ciar et al. [11]. Ten healthy colonies of *H. itama* were used alternately in the two experiments. All of the colonies have a queen, layers of brood cells, and honey pots. Repeated measure design was applied in this study. Each colony was used not more than three times throughout the experiments. 

#### 2.2.1. Experiment 1: Sugar Concentration Preference

Four feeder stands were placed 1 m around the trial bee colony following Ciar et al. [13] (Figure 1). The four feeders were placed in four cardinal directions to the colony, i.e., north, south, east and west. The height of the feeders was set up according to the height of the bee colony’s entrance tube, which was about 1 m from the ground. 

The trial bee colony was moved from the rearing site to the experimental area a day before each trial was conducted to familiarize the bees to the experimental environment. During the actual trial day, the trial colony was presented with four different sucrose water concentrations (*w*/*w* %) (0%, 15%, 35%, and 50%) (HmbG^®^ Chemicals, Johor Bahru, Malaysia). Four Petri dishes were placed on the four different feeder stands. One Petri dish contained 15 mL of saline water (0%) and the other three Petri dishes contained 15-mL sugar (sucrose) solutions of 15%, 35%, and 50% concentration. Each Petri dish was layered with a cotton pad to prevent the stingless bee from drowning in the Petri dish. The solutions in the Petri dishes were refilled every three hours so that the solution (food resource) was always sufficient for stingless bee to forage. The placements of Petri dishes were also rotated clockwise every three hours to reduce bias. In total, 10 colonies were used in Experiment 1.

A picture of bees visiting each feeder were taken three times every hour for 11 h using a digital camera (Canon IXUS 185, Canon, Tokyo, Japan) and the number of bees that landed on the feeders were counted using ImageJ 1.32j software (Wayne Rasband, National Institute of Health, Bethesda, MD, USA). Physical parameters such as temperature (°C) and relative humidity (Rh, %) were recorded during each experiment. The physical parameters were recorded using a 3-in-1 hygro-thermo-anemometer. 

#### 2.2.2. Experiment 2: Foraging Distance 

**Figure 2:**
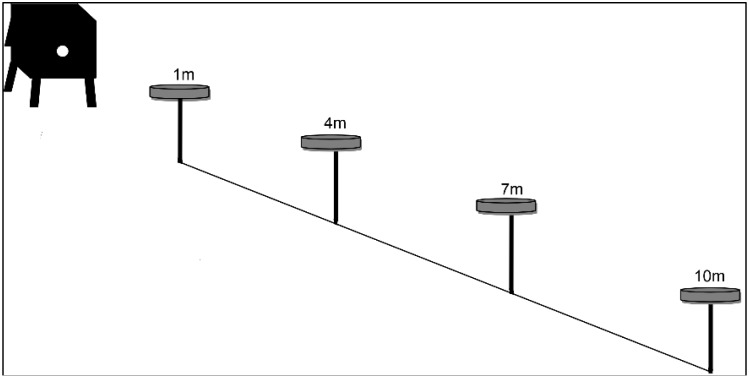
The experimental set-up for Experiment 2 was modified from the method used in Ciar et al. [11]. Four Petri dishes that contained 50% sugar (sucrose) concentrations were placed on the feeder stands at four different distances (one meter, four meters, seven meters, and 10 m) in front of the colony entrance. The grey dot on the beehive represents the entrance of the bee colony. The colony entrance and feeder stands were placed facing east toward the sunrise during the actual experimental trials.

Experiment 2 began two weeks after the last trial in Experiment 1 was conducted to reduce the bees’ memory of previous experiments. The preferred sugar solution from the result in Experiment 1 was used in Experiment 2, which had the 50% sugar solution concentration. Fifteen mL of the 50% sugar solution in Petri dishes were placed on feeder stands aligned at four different distances. Feeders were placed at distances of one meter, four meters, seven meters, and 10 m from the trial colony (Figure 2). The number of bees visiting the feeders was recorded three times per hour for 12 h.

### 2.3. Statistical Analysis

The Shapiro–Wilk test was conducted to analyze the normality of the data. We then performed the Kruskal–Wallis test followed by all of the pairwise multiple comparisons using the Mann–Whitney U-test with Bonferroni correction to analyze the difference in the number of stingless bee visiting the feeders at different concentrations in Experiment 1, and in the number of stingless bee visiting the feeders at different distances in Experiment 2. To analyze the difference in the number of bees visiting the feeders at different times, we divided the foraging times into three time bands: i.e., morning (MR, from 07:00 h until 10:40 h; midday (MD, from 11:00 h until 14:40 h) and late afternoon (LA, from 15:00 h until 18:00 h). We grouped the data of the three time bands to reduce the variance and allow us to conducted general linear modeling (GLM) to determine the difference in the number of bees visiting the different feeders at different time bands. The final GLM for Experiment 1 included sugar concentration, time bands, and the interaction between concentration and time bands as predictor variables. For Experiment 2, the full GLM included distance, time bands, and the interaction between distance and time bands as predictor variables. For foraging pattern analysis, we pooled the number of bees visiting all of the feeders in each hour and conducted the Kruskal–Wallis test, followed by all of the pairwise multiple comparisons. We assumed that the hourly data were independent because the return time for stingless bees has been shown to be about 18 min [22]. A Spearman’s rank-order correlation was run to determine the relationship between the number of bees visiting feeders and the abiotic factors (temperature and relative humidity). All of the analyses were conducted using SPSS (IBM SPSS Statistics for Windows, Version 24.0. Armonk, NY, USA: IBM Corp.).

## 3. Results

### 3.1. Sugar (Sucrose) Concentration Preference

**Figure 3:**
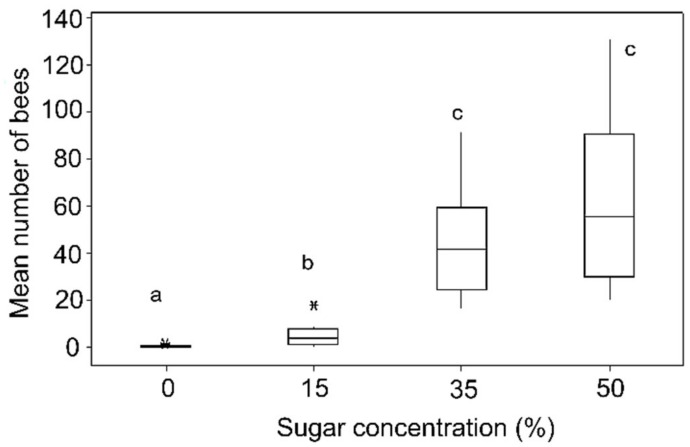
Mean number of *Heterotrigona itama* that visited the four different sucrose concentrations: 0%, 15%, 35%, and 50%. The box demonstrates the interquartile range (IQR), the horizontal line in the box represents the median value of the data. The Whiskers extend represent the maximum and minimum value of the data, excluding outliers. Note that the same letter indicates no significant difference (Kruskal–Wallis test followed by all of the pairwise multiple comparisons, *p* > 0.05, N = 10 trials for all of the variables).

**Figure 4:**
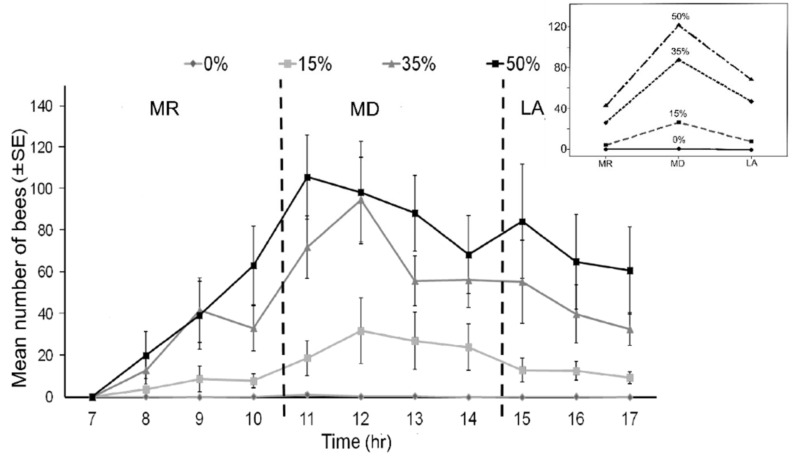
The mean number of stingless bee, *Heterotrigona itama,* that visited the different sugar concentrations (0%, 15%, 35%, and 50%) at different times within an 11-h period. The 11 h of the experiment were divided into three time categories, i.e., morning (MR; 7:00 h–10:40 h); midday (MD; 11:00 h–14:40 h); and late afternoon (LA; 15:00 h–17:40 h). Inset: The interaction plot of the mean number of bees visiting feeders with different sucrose concentrations across the three time categories.

There was a significant difference in the number of stingless bee visiting the feeders containing four different sugar concentrations (Kruskal–Wallis test H_(2)_ = 15.57, *p* < 0.01; Figure 3). Further analysis revealed that this significant difference was within 0% among all of the concentrations and within 15% between the 35% and 50% concentrations. However, the number of bees visiting the 35% and 50% sugar concentrations was not significantly different (*p* > 0.05; Figure 3). The number of bees visiting the feeders with different concentrations in different time bands (i.e., morning, midday, and late afternoon) was significantly different (GLM; F_2108_ = 10.67, *p* < 0.001; Figure 4), but the interaction between time band and sugar concentration was not significant (GLM; F_6108_ = 2.04, *p* = 0.067; Figure 4). This indicates that although the number of bees exploiting the 35% and 50% sugar concentration feeders was higher throughout the day, they seem to be collecting more from the 50% sugar concentration sample during midday (Figure 4, *p* < 0.05).

### 3.2. Food Distance

**Figure 5:**
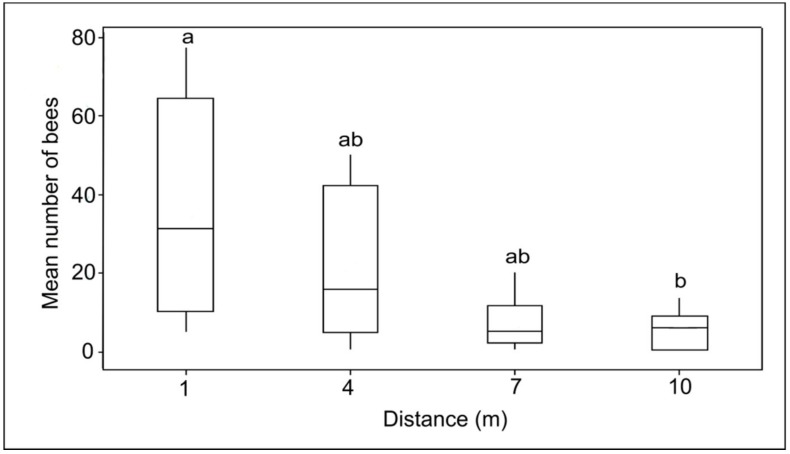
Mean number of *Hetotrigona itama* visiting feeders at different distances: 1 m, 4 m, 7 m, and 10 m. The boxes show the interquartile range (IQR); the horizontal line in the box represents the median value of the data. Whiskers are lines running outside of the box to the maximum and minimum values of the sample. The same letter indicates no significant difference, (Kruskal–Wallis test, *p* > 0.05, N = 10 trials).

**Figure 6:**
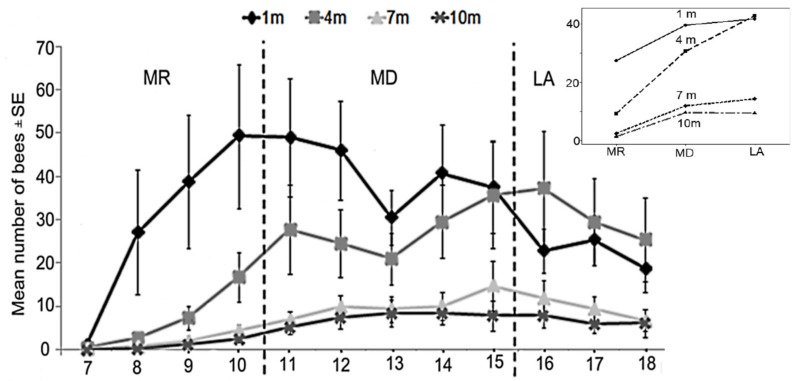
The mean number of *Heterotrigona itama* visited feeders at different distances (1 m, 4 m, 7 m, and 10 m). The time of visitation were categorized into: morning (MR, 7:00–10:40 h), midday (MD, 11:00–14:40 h), and late afternoon (15:00–18:40 h). Inset: The interaction plot of the mean number of bees visiting feeders at different distances across the three time categories.

There was a significant difference in the number of bees visiting feeders at different distances (Kruskal–Wallis test; H_(3)_ = 12.62, *p* = 0.006; Figure 5). Further analysis revealed that only the feeder that was placed one meter from the beehive showed a significant difference compared with the feeder placed 10 m from the beehive (*p* = 0.01). There was also a significant difference in the number of bees visiting the feeders at different distances across the three time bands (i.e., morning versus midday versus late afternoon; GLM; F_3108_ = 11.14, *p* < 0.01; Figure 6). Bees visiting the feeder placed one meter from the hive were much higher in number during the midday and late afternoon (*p* < 0.05) periods, but there was no effect of interaction between the number of bees visiting feeders at different distances across the three different time bands (GLM; F_6108_ = 0.60, *p* = 0.731; Figure 6).

### 3.3. Foraging Pattern

**Figure 7:**
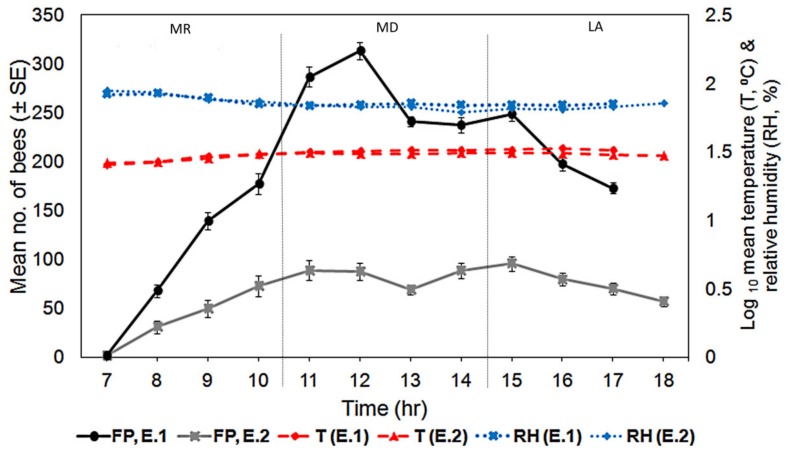
Foraging pattern (FP) of *Heterotrigona itama* based on the data collected in Experiment 1 (E.1; different concentration) and Experiment 2 (E.2; equal concentration). The experimental period for Experiment 1 was 11 h, while it was 12 h for Experiment 2 h at field. The red dashed lines indicate the mean temperature (T), and the blue dotted lines indicate the relative humidity (RH) of E1 and E2. (E.1: MR; T: 30.45 ± 0.43 °C, RH: 71.28 ± 2.24%; MD: T: 31.22 ± 0.83 °C, RH: 70.25 ± 2.87%; LA; T: 31.11 ± 0.82 °C, RH: 71.04 ± 3.42%. E.2: MR; T: 28.00 ± 0.41 °C, RH: 74.13 ± 1.44%; MD: T: 30.54 ± 0.48 °C, RH: 70.88 ± 1.47%; LA; T: 30.51 ± 0.52 °C, RH: 74.61 ± 1.18%).

Based on the number of foraging bees in the first experiment (Experiment 1: sugar concentration preference), the number of foragers that visited the different sugar concentration feeders (0%, 15%, 35%, and 50%) increased significantly from 07:00 h to reach a peak at noon (H_(10)_ = 30.40, *p* < 0.01, Figure 7). In order to examine the effect of distance of food source from the nest, the same pattern of foraging was observed in the second experiment; the number of bees exploiting the food sources at different distances increased from 07:00 h and peaked at 11:00 h (H_(11)_ = 39.53, *p* < 0.0, Figure 7). In both experiments, the number of foragers dropped after midday (from 12:20 h to 13:00 h). The number of foraging bees continued to decline until the late afternoon (17:40 h) in Experiment 1. In Experiment 2, the number of bees exploiting the food sources gradually increased again from 14:00 h until 15:00 h before cessation from 16:00 h until dusk. There was a slightly insignificant correlation between the number of bees visiting the feeders and temperature (r_s(9)_ = 0.573, *p* = 0.066), and also no correlation with relative humidity (r_s(9)_ = −0.257, *p* = 0.096) in Experiment 1, whereby food sources were available near the hive. In Experiment 2, where all of the food sources were placed at different distances from the hive, the result showed that there was a strong positive correlation between the number of bees visiting feeders and temperature (r_s(10)_ = −0.953, *p* < 0.01) and a negative correlation with relative humidity (r_s(10)_ = −0.902, *p* < 0.01).

## 4. Discussion

We looked into the food exploitation of *Heterotrigona itama* by presenting different concentrations of sucrose solutions and placing the same sugar solutions at different distances from the nest. We then compared the foraging pattern of the stingless bee between the two experimental set-ups.

Food with a 50% sugar concentration was expected to be preferred by *H. itama* as compared with lower sugar concentrations [18]. However, our results showed that the number of *H. itama* foragers exploiting the 35% and 50% of food concentrations were not different, indicating that this species may not differentiate between flowers with nectar concentrations of 35% and above. Bees are known to choose and exploit the most profitable food sources even though both of the food sources are found concurrently [23]. Moreover, for Meliponini bees, the higher nectar concentrations ranging between 20–60% are preferred [16]. Preference for high sugar nectar may be determined by nectar-drinking habits. While certain species of bees may drink the nectar via active suction, many other species employ a viscous dipping technique [24]. In nature, nectar with low concentration (35% and lower) is suitable for the active suction drinker (for example the orchid bee), while a higher nectar concentration (50% to 60%) is better suited to the viscous dipping types [24]. Since *H. itama* employs a viscous dipping technique, a much higher nectar concentration would be advantageous. However, bees also have their optimal nectar concentration preference according to their body sizes [16,25]. Larger bees prefer higher concentrations of nectar while smaller bees favor lower concentrations of sugar in nectar [25]. For example, *Trigona muzoensis,* with a 6-mm body size*,* prefers nectar sugar concentration of 30% and its optimal concentration is 60%, while the 9-mm *Melipona beechei* has an optimal nectar sugar concentration preference of 65%. In our study on *H. itama*, with its body size of about 5 mm, the preferred sugar concentrations are 35% and 50%, and the optimal concentration for this species most likely would be higher than 50%. Moreover, our result also showed that highest number of *H. itama* exploited the highest sugar concentration (50%) during the midday, as the environmental temperature was rising. Studies by Kajobe [11] and Macías-Macías [21] on different species of stingless bee also found a positive correlation between the environmental temperature and the nectar concentration taken by stingless bees. This may be a strategic decision made by the bees, since dipping into high sugar concentration enables them to collect much of the sugar water in a short time [24,26], and hence they could save their energy while foraging in the heat.

The body size of the bee may also affect its foraging distance, with stingless bees of different sizes having varying abilities to forage far from their nests [22]. For example, *M. mandacaia*, a small forager, tends to travel only short distances, whereas the larger *M. mandacaia* can wander further when foraging [25]. In the case of *H. itama*, body size may be a reason for this species preferring to exploit food sources that are nearer (1 m away) than those from feeders that are further (10 m) away. The recruitment of nestmates to a food source that is located a considerable distance away is also problematic, because the journey is energy-costly, while not always profitable or worth the effort [27]. Nevertheless, under certain circumstances in nature, bees may travel long distances from their hives to seek food [28].

The foraging activities of bees can be influenced by many factors such as temperature, food source availability, and colony condition. Although almost all of the species of diurnal bees are active from early morning, with their foraging activity usually peaking around 11:00 h to 12:00 h [5,29,30], we found that food reward may change this pattern for *H. itama*. In this study, when food with different sugar concentrations were presented near the colonies (1 m around the colonies), the foraging activity of the stingless bees increased from morning until about 12:00 h, and slowly ceased after that. High numbers of foragers visited the feeders when the food sources were near the hive and their activity showed no correlation with abiotic factors. However, when higher concentrations of food sources were presented to the colonies at different distances, the total number of bees visiting all of the feeders was smaller, with the majority of the bees visiting feeders in the morning between 10:00–12:00 h, and in the afternoon between 14:00–16:00 h. The bees’ foraging activity under the second experiment also showed a significant correlation with abiotic factors. In other species of stingless bees, two peaked foraging hours have been recorded for *Trigona iridipennis*, which showed a similar pattern with our study, whereby the first peak foraging hour was in the morning (09.00 h to 11:00 h), and the second was in the late afternoon (15:00 h–16:00 h) [3]. The insignificance correlation value between the foraging pattern and abiotic factors in Experiment 1 was small (*p* = 0.066). Hence, our results suggest that when food sources were abundant near the beehive, the effect of abiotic factors on the bees’ foraging activity may be reduced. Our results also showed a sharp decline in the foraging activity during midday from 12:00 h until 13:00 h, which was probably because of the high temperature (exceeding 30 °C). Foraging in high environmental temperature requires more energy and may cause dehydration among the foragers.

The information gained from this study would be helpful to agriculturists and stingless beekeepers in deciding the position of beehives in relation to food sources on their farms as well as in crop fields [13]. This study can also enhance stingless beekeepers’ knowledge of the specific behavior of *H. itama,* thus helping them sustain the bee colonies.

## 5. Conclusions

Our study showed that *H. itama* prefers nectar with a sugar concentration that is higher than 35%. As the environmental temperature rises, many bees prefer exploiting the highest sugar concentration. While the availability of food near the beehives enables many foragers to exploit the food sources, foraging activity drops as the environmental temperature increases toward midday from 12:00 h until 13:00 h. From the early afternoon, active foraging resumes where food with high sugar concentration (better reward) is abundant. Overall, our results show that nectar concentration and food distance influence both the number of bees exploiting food resources and the overall foraging pattern of *H. itama*. The results from this study can help stingless beekeepers understand the behavior of stingless bees, so that they can make good decisions about the arrangement of hives in relation to distances to food sources, especially in the small crop field.
